# Genomic approach to explore altered signaling networks of olfaction in response to diesel exhaust particles in mice

**DOI:** 10.1038/s41598-020-74109-6

**Published:** 2020-10-12

**Authors:** Su Ji Kim, Nahyun Kim, So Hyeon Park, Hyun Soo Kim, Jae-Jun Song, Bu-Soon Son, An-Soo Jang, Moo Kyun Park, Young Rok Seo

**Affiliations:** 1 Department of Life Science, Institute of Environmental Medicine for Green Chemistry, Dongguk University Biomedi Campus, 32 Dongguk-ro, Ilsandong-gu, Goyang-si, Gyeonggi-do 10326 Republic of Korea; 2 Department of Otorhinolaryngology, Head and Neck Surgery, Seoul National University Hospital, Seoul National University College of Medicine, 101 Daehak-ro, Jongno-gu, Seoul, 03080 Republic of Korea; 3 Department of Otorhinolaryngology-Head and Neck Surgery, Korea University College of Medicine, Seoul, South Korea; 4 Department of Environmental Health Science, Soonchunhyang University, Asan, Republic of Korea; 5 Division of Allergy and Respiratory Medicine, Department of Internal Medicine, Soonchunhyang University Bucheon Hospital, Bucheon, South Korea

**Keywords:** Neuroscience, Environmental sciences, Health care, Neurology

## Abstract

Airborne pollutants have detrimental effect on the human body and the environment. Diesel exhaust particles (DEPs) are known to be major component of particulate matter (PM) and cause respiratory diseases and neurotoxicity. However, the effects of air pollutants on the sensory nervous system, especially on the olfactory sense, have not been well studied. Herein, we aimed to explore DEP-induced changes in the olfactory perception process. Olfactory sensitivity test was performed after DEP inhalation in mice. Microarray was conducted to determine the differentially expressed genes, which were then utilized to build a network focused on neurotoxicity. Exposure to DEPs significantly reduced sniffing in mice, indicating a disturbance in the olfactory perception process. Through network analysis, we proposed five genes (*Cfap69*, *Cyp26b1*, *Il1b*, *Il6*, and *Synpr*) as biomarker candidates for DEP-mediated olfactory dysfunction. Changes in their expression might provoke malfunction of sensory transduction by inhibiting olfactory receptors, neurite outgrowth, and axonal guidance as well as lead to failure of recovery from neuroinflammatory damage through inhibition of nerve regeneration. Thus, we suggest the potential mechanism underlying DEPs-mediated olfactory disorders using genomic approach. Our study will be helpful to future researchers to assess an individual’s olfactory vulnerability following exposure to inhalational environmental hazards.

## Introduction

Emission of diesel engine exhaust is a well-known representative cause of ambient air pollution, and diesel engines have been widely used in industrial sectors for transportation, mining, and agriculture^1^. Diesel exhaust particles (DEPs) consist of an elemental carbon core, adsorbed organic compounds such as polycyclic aromatic hydrocarbons, and small amounts of nitrate, sulfate, and other metals^2^. Most DEPs are less than 1 μm in diameter^3^. Research-based evidence has shown that DEPs can exert adverse effects on human health, especially in respiratory and cardiovascular systems, given that they contain toxic substances and are very small in size^4–7^. Furthermore, there is increasing evidence to show that various air pollutants including DEPs are correlated with neurotoxicity^8–10^.

The olfactory system is one of the most developed and sensitive molecular-sensing organ systems. It is composed of the olfactory epithelium (OE) that is made up of olfactory sensory neurons (OSNs) inside the nasal cavity, and olfactory bulb (OB) in the forebrain^11^. Once inhaled air containing odor molecules enter the nasal cavity, OSN receptors, the first part where the entered molecules encounter, detect the odorants. Then, OSNs initiate sensory transduction that spreads the electric response into the glomerulus in the olfactory bulb through axonal guidance^12,13^. This series of process is called olfactory perception. Sniffing is mechanical sensing by inhalation via the nose and is known as essential behavior for olfactory perception^14^. Indeed, olfactory dysfunction with reduced number of sniff times is observed in patients with neurological diseases^15,16^.

Olfactory impairment can be attributed by various factors such as aging, air pollution, airway allergy, and exposure to toxic chemicals. Especially in terms of air pollution, Ueha et al. demonstrated that cigarette smoke decreases the number of mature OSNs by up-regulation of tumor necrosis factor (*Tnf*) and interleukin 6 (*Il6*)^17^. Further, Cheng et al. showed that nanoscale particulate matter (nPM) induces oxidative stress and inflammatory responses in the OE^18^. However, DEP-mediated dysregulation of olfaction has been poorly studied. A recent study using a rodent model suggested that maternal exposure to DEPs affected neurodevelopment by nanoparticles penetration into the fetal olfactory structure^19^.

In this study, we aimed to explore alterations of olfactory function in response to DEP exposure in mice. We conducted olfaction sensitivity tests after DEP inhalation in mice. To understand the signaling network associated with DEP-induced olfactory dysfunction, pathway analysis was also performed with differentially expressed genes (DEGs) from the microarray data. This would provide a better understanding for further studies on the mechanism of DEP-mediated olfactory disturbance.

## Results

### Exposure to DEPs decreases olfactory sensitivity in mice

**Figure 1:**
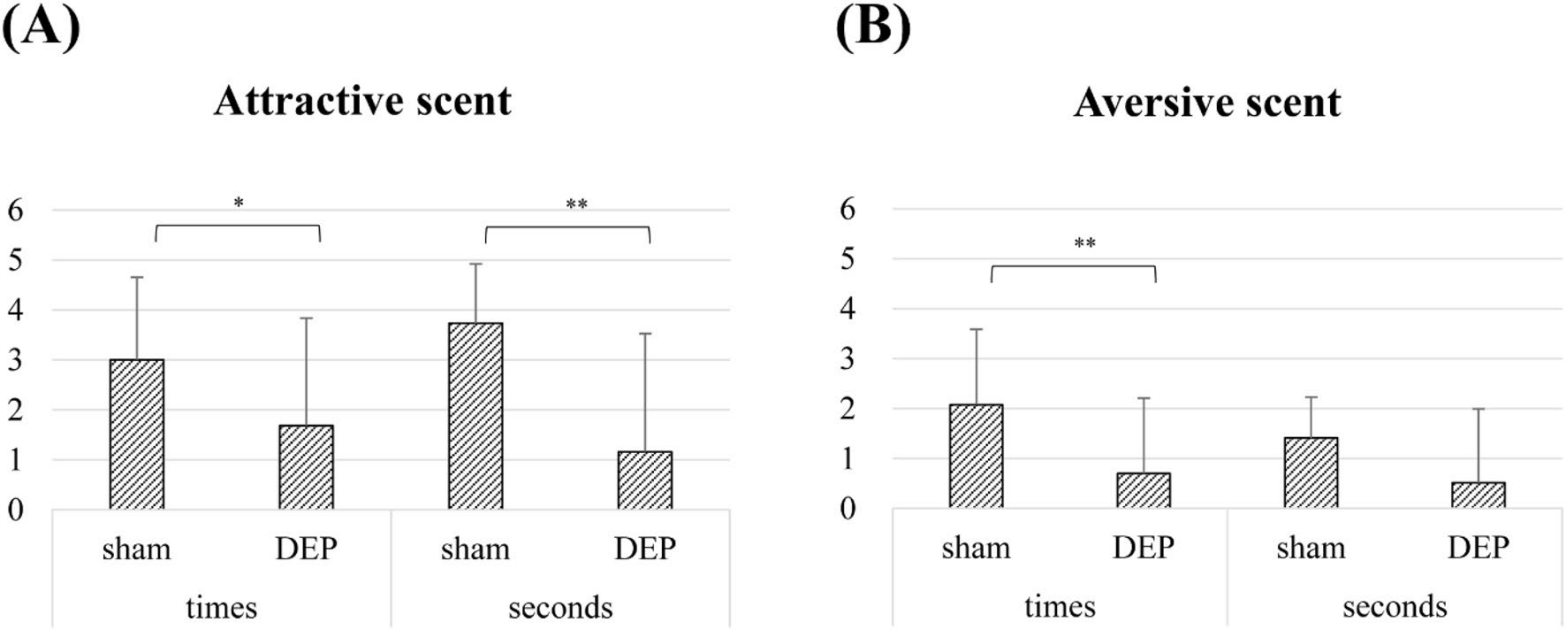
Exposure to DEPs decreases olfactory sensitivity in mice. (**A**) Sniffing times and seconds were measured using 10% peanut butter solution as the attractive scent. Both times and seconds significantly decreased after DEP inhalation. (**B**) Sniffing times and seconds were measured using 10% trichloroacetic acid solution as the aversive scent. The number of sniffing significantly decreased in mice that inhaled DEPs. Statistical significance was indicated as * for *p*-value ≤ 0.05 and as ** for *p*-value ≤ 0.01.

To assess the harmful effect of DEPs on olfactory perception, sensitivity test was conducted by counting the sniffing times and seconds in mice. Additionally, we used both attractive and aversive scents to distinguish the difference in behavior depending on the exposure to preferred and non-preferred odorants. In the group with aversive scent, sniffing times and seconds are generally lower than group with attractive scent. As a result, after DEPs exposure for four weeks, the frequency and duration of sniffs decreased significantly in both attractive and aversive scent except for the seconds in aversive odorant only (Fig. 1). This suppression in sniffing suggests that inhalation of DEPs may lead to decreased ability of olfactory perception, because sniffs are an important factor in evaluating the ability to recognize odorants.

### DEPs alter the expression of genes involved in olfactory reception and sensory transduction networks

We screened genes whose expressions were altered in response to DEP exposure, through microarray data according to strict standards with a *p*-value ≤ 0.01 and |fold change| ≥ 2. In all, 397 up-regulated genes and 134 down-regulated genes were differentially expressed by DEP exposure in the mouse nasal tissue. A total of 558 DEGs were input into the software (Table S2).

Based on the results of the behavior test, we could conclude that DEPs disturb the olfactory perception process. Although a number of air pollutants are known to induce olfactory disorders, the exact mode of action of DEP-mediated olfactory dysfunction remains unclear. To explore the potential olfactory-specific mechanism altered by DEPs, we utilized the Pathway Studio software which enabled analysis of gene expression data to establish a signaling network composed of interactions between genes, cell processes, and diseases.

**Figure 2:**
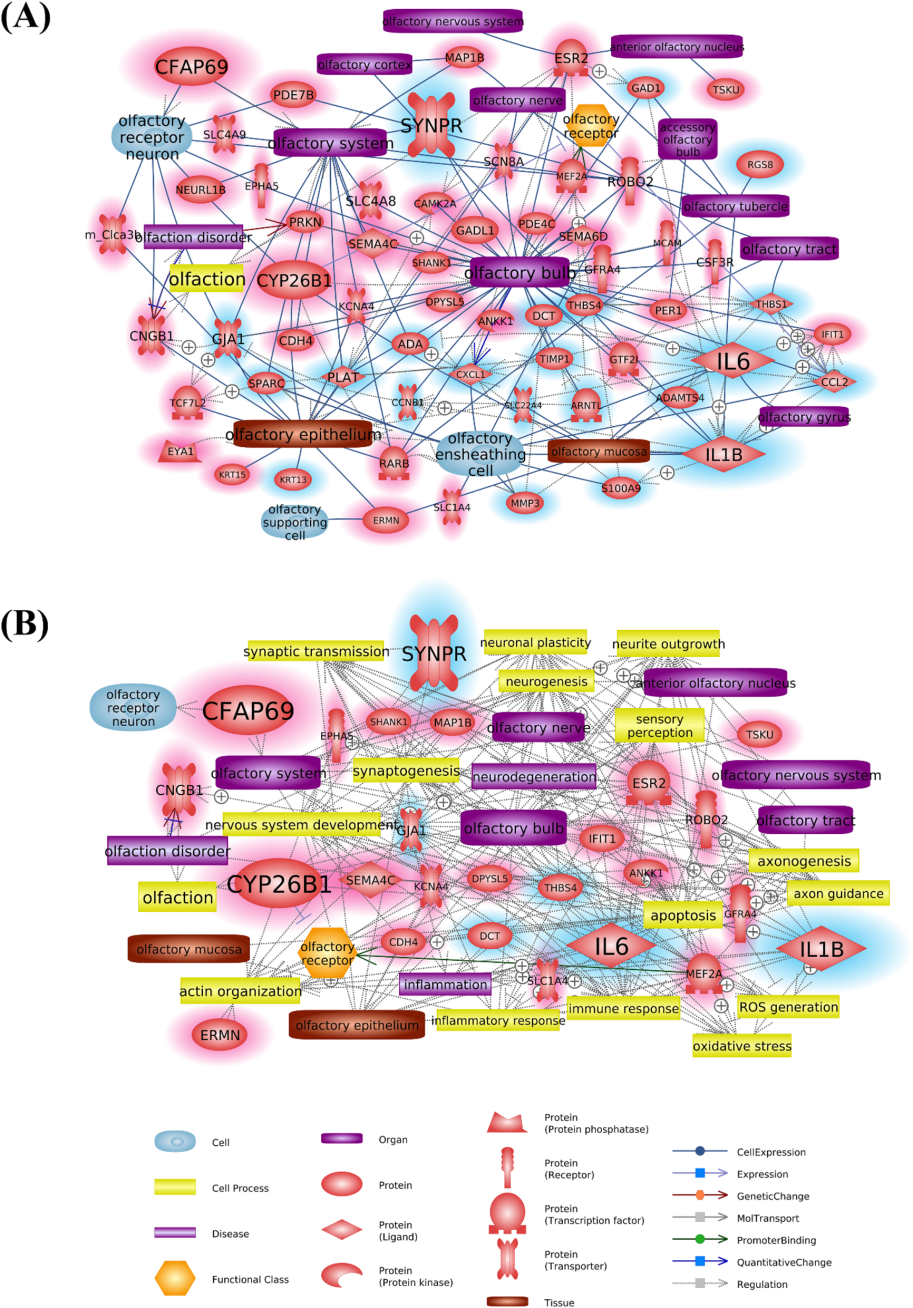
Signaling networks suggesting biological interactions of olfactory system- associated genes following DEP exposure. (**A**) A network consisting of connections between olfactory factors and significantly altered genes. Olfactory factors include various types of cell, tissues, and organs. (**B**) Expanded network with cell process and disease of olfactory-associated genes. Genes highlighted in red and blue color indicate the pattern of increased and decreased expression, respectively. The major contributing genes in the network considering the number of references and connectivity rate are marked lager than other entities. The legend for entities is at the bottom of the figure.

To construct olfaction-specific network, we first connected the identified DEGs with olfactory factors that include various types of cell, tissues, and organs such as olfactory supporting cell, olfactory mucosa, and OB, etc., provided by the Pathway Studio (Fig. 2A). The information of associations between DEGs and olfactory factors is summarized in Table 1. Among these entities, the main components of the olfactory system such as OB, OE, and ensheathing cells showed the highest number of connections in the network, implying that DEPs are involved in the genetic regulation of these factors. Among the DEGs, cilia and flagella associated protein 69 (*Cfap69*) and cytochrome P450 family 26 subfamily B member 1 (*Cyp26b1*) were up-regulated genes, and interleukin 1 beta (*Il1b*), *Il6*, and synaptoporin (*Synpr*) were down-regulated genes, and suggested to be the main contributors in the network considering the number of references and connectivity with other entities. Interestingly, pro-inflammatory cytokines encoded by *Il1b* and *Il6* were shown to be significantly inhibited by DEPs.

**Table 1:** List of genes connected with olfactory factors.

Entity	Type	Relation	Gene
Accessory olfactory bulb	Organ	Cell expression	GAD1, PER1
		Regulation	ROBO2
Anterior olfactory nucleus	Organ	Cell expression	ESR2, TSKU
Olfactory bulb	Organ	Cell expression	ANKK1, ARNTL, CAMK2A, CCL2, CCNB1, CDH4, CSF3R, CXCL1, DCT, CPYSL5, EPHA5, ESR2, GAD1, GADL1, GFRA4, GJA1, GTF2I, IFIT1, IL1B, IL6, KCNA4, MCAM, PDE4C, PER1, PLAT, PRKN, RARB, ROBO2, SCN8A, SEMA4C, SEMA6D, SHANK1, SLC4A8, SYNPR, THBS4, TIMP1
		Regulation	CYP26B1, GJA1, MCAM, MEF2A, PRKN, ROBO2, THBS1
		Quantitative change	CXCL1
Olfactory cortex	Organ	Cell expression	IL1B, MAP1B
Olfactory ensheathing cell	Cell	Cell expression	CCL2, CXCL1, GJA1, IL1B, IL6, MMP3, ROBO2, S100A9, SLC1A4, SPARC
		Molecular transport	MMP3
		Regulation	RARB
Olfactory epithelium	Tissue	Cell expression	CYP26B1, ERMN, ESR2, GJA1, KCNA4, PLAT, RARB, ROBO2
		Regulation	EYA1, IL1B, IL6, KCNA4, PLAT
Olfactory gyrus	Organ	Cell expression	IL6
Olfactory mucosa	Tissue	Cell expression	IL1B, IL6
Olfactory nerve	Organ	Cell expression	MAP1B, ROBO2, SCN8A
		Regulation	ROBO2
Olfactory nervous system	Organ	Cell expression	ESR2
Olfactory receptor neuron	Cell	Cell expression	CFAP69, CNGB1, CYP26B1, EPHA5, GJA1, m_Clca3b, PDE7B, ROBO2
		Regulation	CFAP69
Olfactory supporting cell	Cell	Cell expression	ERMN
Olfactory system	Organ	Cell expression	ADA, CDH4, CFAP69, GJA1, MAP1B, NEURL1B, PLAT, RARB, ROBO2, SEMA4C, SLC4A8, SLC4A9, SPARC, SYNPR
		Regulation	CFAP69, MAP1B, PLAT, PRKN
Olfactory tract	Organ	Cell expression	PER1, ROBO2, THBS1
Olfactory tubercle	Organ	Cell expression	ESR2, GAD1, IL1B, PDE7B, RARB, RGS8

**Figure 3:**
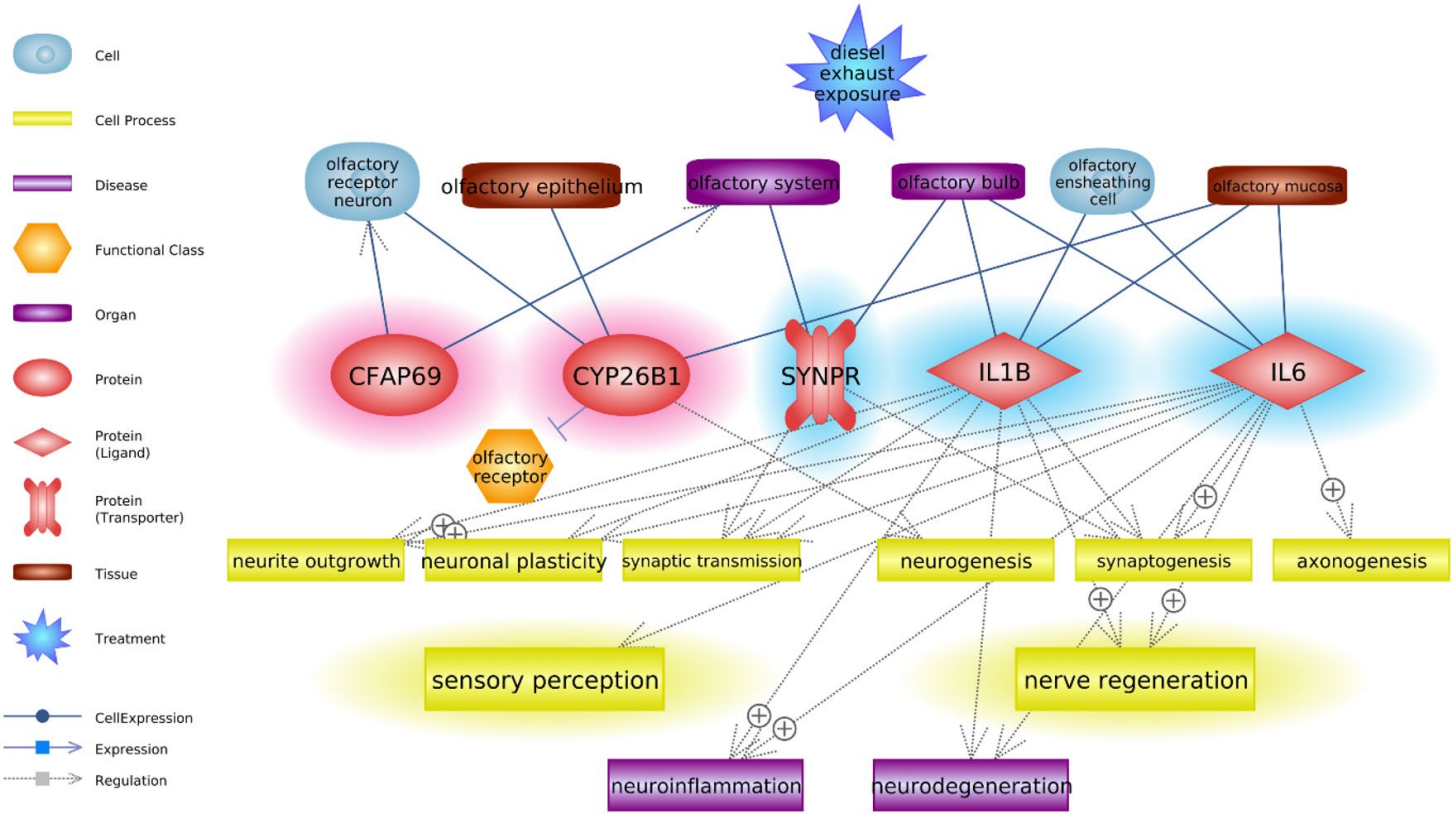
Schematic pathway for DEP-mediated olfactory dysfunction. Simplified signaling network of major genes altered by DEPs. Up- and down-regulated genes lead to olfactory disorders caused by neurotoxicity through inhibition of sensory perception and nerve regeneration. Genes highlighted in red and blue color indicate the pattern of increased and decreased expression, respectively. Main cell processes are highlighted in yellow color. The legend for entities is on the left side of the figure.

To determine altered biological functions depending on gene expression change induced by DEP exposure, the expanded network was analyzed. This includes cellular processes and diseases regulated by DEGs and functionally associated with olfactory factors (Fig. 2B). The gene list in connection with the cell process, functional class, and disease is indicated in Table 2. Expanded cellular processes were mainly involved with apoptosis, nervous system development, neurite outgrowth, axon guidance, and inflammatory response. Consistent with the result of the behavioral test, we found that DEP-exposure would have the greatest impact on the nervous system, because most DEGs linked to olfactory factors (Fig. 1A) were associated with neuronal plasticity, synaptic transmission, and axonogenesis. Moreover, intracellular roles of the abovementioned key genes were analyzed to explore their biological functions in the network and were schematized in Fig. 3. Although there was no association of *Cfap69* with cell process or disease as it has been rarely studied, the connectivity ratio was the highest in the pathway. *Cyp26b1* appeared to disturb the olfaction process through inhibition of the olfactory receptor. Moreover, *Synpr* expressed in the OB was shown to regulate synaptic transmission. Both *Il6* and *Il1b* were associated with similar cell processes including inflammatory response, neuronal plasticity, and neurite outgrowth.

**Table 2:** List of genes related to cell process, disease, and functional class altered by DEP exposure.

Entity	Type	Relation	Gene
Actin organization	Cell process	Regulation	DPYSL5, EPHA5, ERMN, ESR2, GJA1, IL1B, IL6, MAP1B, MEF2A, ROBO2
Apoptosis	Cell process	Regulation	ANKK1, CYP26B1, DCT, ESR2, GFRA4, GJA1, IFIT1, IL1B, IL6, MAP1B, MEF2A, ROBO2, THBS4
Axon guidance	Cell process	Regulation	CDH, DPYSL5, EPHA5, ESR2, MAP1B, ROBO2, SEMA4C
Axonogenesis	Cell process	Regulation	CDH4, DPYSL5, EPHA5, IL6, MAP1B, MEF2A, ROBO2
Immune response	Cell process	Regulation	DCT, DPYSL5, ESR2, GJA1, IFIT1, IL1B, IL6, KCNA4, MEF2A, ROBO2
Inflammatory response	Cell process	Regulation	ESR2, GJA1, IL1B, IL6, MEF2A, THBS4
Nervous system development	Cell process	Regulation	CDH4, DPYSL5, EPHA5, ESR2, GJA1, IL1B, IL6, MAP1B, MEF2A, ROBO2, SEMA4C, SLC1A4, SYNPR, THBS4, TSKU
Neurite outgrowth	Cell process	Regulation	CDH4, DPYSL5, ESR2, GFRA4, GJA1, IL1B, IL6, MAP1B, MEF2A, ROBO2, THBS4, TSKU
Neurogenesis	Cell process	Regulation	CYP26B1, DPYSL5, EPHA5, ESR2, GJA1, MAP1B, MEF2A, ROBO2
Neuronal plasticity	Cell process	Regulation	EPHA5, ESR2, GJA1, IL1B, IL6, MAP1B, MEF2A, THBS4
Olfaction	Cell process	Regulation	CNGB1, ESR2
Oxidative stress	Cell process	Regulation	DCT, ESR2, GJA1, IL1B, IL6, MEF2A
ROS generation	Cell process	Regulation	ESR2, GJA1, IL1B, IL6, MEF2A
Sensory perception	Cell process	Regulation	ESR2, GJA1, IL6
Synaptic transmission	Cell process	Regulation	ESR2, GJA1, IL1B, IL6, KCNA4, MAP1B, MEF2A, SHANK1, SYNPR, THBS4
Synaptogenesis	Cell process	Regulation	EPHA5, ESR2, GJA1, IL1B, IL6, MAP1B, MEF2A, ROBO2, SHANK1, SYNPR, THBS4
Inflammation	Disease	Regulation	ESR2, GJA1, IL1B, IL6, THBS4
Neurodegeneration	Disease	Regulation	ESR2, IL1B, IL6, MAP1B
Olfaction disorder	Disease	Genetic change	CNGB1
		Quantitative change	CNGB1
		Regulation	CNGB1
Olfactory receptor	Functional class	Expression	CYP26B1, DPYSL5, EPHA5, ESR2, GJA1, MAP1B, MEF2A, ROBO2
		Promoter binding	MEF2A

### Validation of the key genes suggesting DEPs-mediated olfactory dysfunction

**Figure 4:**
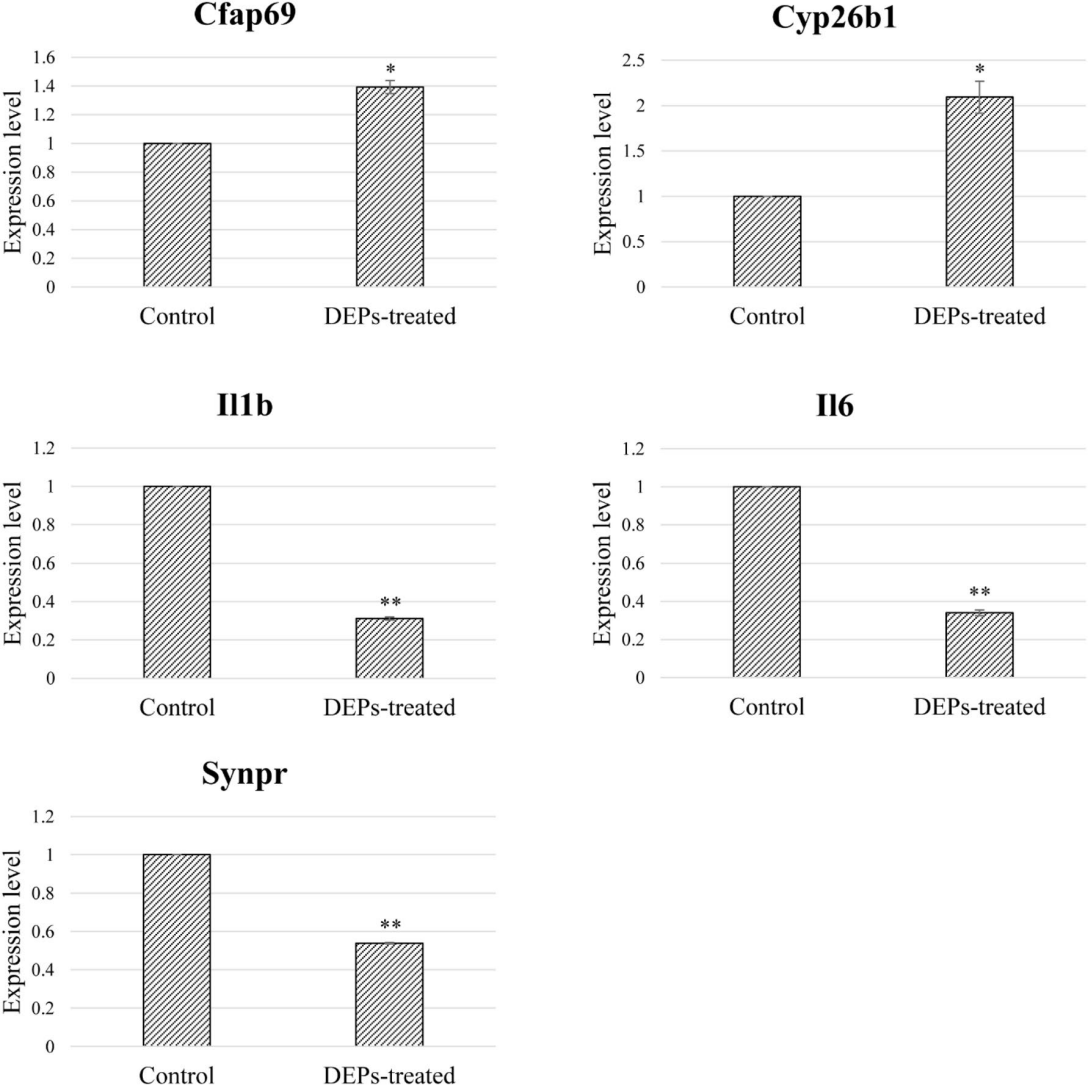
Validation of major gene expressions involved in DEP-mediated olfactory dysfunction pathway. The mRNA expression level of five key genes was verified using qRT-PCR. Each gene has an expression pattern consistent with the microarray result. Error bars represent means ± standard deviations. Statistical significance was indicated as * for *p*-value ≤ 0.05 and as ** for *p*-value ≤ 0.01.

To elucidate the expression trend of the suggested five key genes, we carried out qRT-PCR using the same nasal tissue used in microarrays. As indicated in Fig. 4, two up-regulated and three down-regulated genes were shown to be expressed in a similar manner to the microarray data.

## Discussion

There are increasing concerns about the diseases caused by air pollutants such as DEPs. Exposure to DEPs has been proven to be associated with respiratory tract diseases including asthma and chronic obstructive pulmonary disease^20–22^. Although neurodegenerative diseases such as Alzheimer’s and Parkinson’s disease have also been found to be correlated with air pollutants, the exact mechanisms of neurological damage induced by DEPs is still unclear. DEPs also have deleterious effects not only on the olfactory system but also on other sensory systems such as visual and auditory. Previous studies have reported that administration of DEPs caused disruption of ocular structure in mice and increased the inflammatory response in human middle ear epithelial cells^23,24^. In this regard we studied the effect of sub-acute inhalation of DEPs on sensory impairment, especially on olfactory system using mice model.

We conducted the olfactory sensitivity test by measuring the frequency and duration of sniffs. Sniffs are regarded as necessary acts for olfactory cognitive process to occur through OSN-mediated sensory transduction^14,25,26^. It was determined that an absence of mechanical stimulus led to the absence of an olfactory sensation^27^. Accordingly, whether the olfactory perception process works well can be assessed by measuring change in sniff behavior. Figure 1 shows that DEP exposure significantly reduced the sniffing frequency and duration. Based on this result, we hypothesized that DEPs would impair the olfactory perceptive capacity by interfering with the olfactory transduction process. In the present study, we explored putative biological mechanisms of the olfactory perception process through network analysis of DEGs following DEP inhalation.

To observe changes in gene expression depending on sub-acute DEP inhalation, we performed microarray with RNA extracted from the nasal tissues including nasal septum and turbinate in mice. Because the nasal cavity contains the OE where OSN-mediated olfactory transduction takes place, it is considered to be more appropriate than the OB for identification of genes that play an essential role in the olfactory cognitive process. Moreover, we re-screened DEGs from the nasal tissue to obtain more olfactory-specific genes by linking them with olfaction-related entities provided by the Pathway Studio database. Consequently, a reliable olfactory-related gene set was generated (Table 1).

Figure 2A shows interactions between DEGs and various olfactory-related factors present in the database. The blue lines that occupy most part of the relations indicate the expression of genes in the olfactory system such as the OB, OE, and OSNs. It is noteworthy that changes in the expression of these genes can lead to modulations of their molecular functions in each organ. We suggested five genes as biomarker candidates for DEP-mediated suppression of olfactory perception through the network analysis: two up-regulated genes—*Cfap69* and *Cyp26b1*—and three down-regulated genes—*Synpr*, *Il1b*, and *Il6*—in response to DEP exposure.

*Cfap69* is mainly enriched in the cilia of OSNs, where olfactory receptors detect odorant molecules. *Cfap69* facilitates the machinery of olfactory transduction to operate at an accurately regulated range of response kinetics to modulate olfactory behavior^28^. Accordingly, high expression of *Cfap69* does not facilitate optimal sensory transduction. *Synpr* encodes for synaptoporin, a membrane protein of synaptic vesicles. It has been reported that expression change of synaptoporin following sensory nerve injury leads to an alternation in the synaptic transmission^29^. Down-regulated *Synpr* would result in lower expression of synaptoporin, causing dysregulation of synaptic transmission in sensory terminals under DEP treatment. Expression change of these two genes by DEPs could interfere with the sensory transduction process, leading to failure of olfactory perception.

The other up-regulated gene, *Cyp26b1*, belongs to the cytochrome P450 family and has retinoic acid (RA)-metabolizing activity. RA, a metabolite of vitamin A, regulates the fate of olfactory progenitor cells, leading to olfactory neuronal production^30^. RA is also an essential regulator of regeneration in the mammalian olfactory pathway^31,32^. Indeed, vitamin A therapy in patients with olfactory impairment has contributed to functional recovery^33^. Accordingly, the up-regulation of *Cyp26b1* would suppress the regenerative process after DEP-induced olfactory damage by increasing RA degradation.

*Il1b* and *Il6* encode well known pro-inflammatory cytokines released from immune cells. Extensive studies have shown that DEPs induce pro-inflammatory cytokines regardless of exposure concentration and duration. Intriguingly, we observed significant downregulation of *Il1b* and *Il6*. This inconsistency might be attributed to abnormal immune function by DEP-mediated damage^34^. *Il1b* and *Il6* play essential roles to promote neuronal regeneration and to regulate sensory functions and processing after nerve injury^35–40^. The significantly altered expression of these two genes might lead to incomplete recovery against DEPs-induced sensory nerve injury by inhibiting nerve regeneration.

Figure 2B illustrates the cellular processes and diseases of olfactory-related genes, which revealed the molecular function of the DEGs in network and potential mechanism of olfactory dysfunction induced by DEPs. Cellular processes related to neuronal function had the most connections, indicating DEP-induced neurotoxicity. Furthermore, the network specifically included diseases related to sensory disturbance, such as inflammation, olfaction disorder, and neurodegeneration. In particular, because olfactory disorder is a major clinical manifestation of neurological diseases^41^, neuroinflammatory and neurotoxic response by DEP inhalation might cause decline of olfactory function. This is in agreement with the roles of the five key genes in inhibiting sensory transduction and nerve damage repair. Through the expanded network analysis, we proposed that DEP-mediated modulation of gene expression in the mouse nasal cavity might lead to disturbance of olfactory perceptual processes including olfactory reception and sensory transduction in OSNs.

Although expression level of crucial genes was validated via qRT-PCR and consistent with the microarray results (Fig. 4), further functional studies are needed to confirm the exact mechanism of DEP-induced olfactory dysfunction. As the complexes of the olfactory system and participating proteins are involved and interact with each other in the odor recognition process, there is a limit to suggesting altered olfactory function by identifying changes in expression of a single gene and subsequent cellular process.

In summary, among the DEGs owing to DEP exposure in the mouse nasal cavity, *Cfap69*, *Cyp26b1*, *Il1b*, *Il6*, and *Synpr* appear to be major contributors in olfactory network and are closely associated with expression in the olfactory system such as OB, OE, and OSNs. Moreover, changes in the expression of these genes may not only control sensory transduction in the olfactory perceptual process via inhibition of axonal guidance and neurite outgrowth but also have a harmful effect on the recovery of olfactory nerve damage by interrupting neuronal regeneration (Fig. 3). Although further studies on the mechanism are required, we believe our study provides a better understanding for DEP-mediated olfactory disorders such as anosmia and hyposmia via DEG-based molecular network analysis. This study might help to improve healthcare system for assessing individual olfactory sensitivity in response to environmentally harmful substances.

## Methods

### Animals preparation and DEP exposure

All animal experiments were conducted according to the guidelines approved by the Institutional Animal Care and Use Committee at Soonchunhyang University Medical School (SCHBC-Animal-2014-013). All experimental protocols were approved by the Institutional Animal Care and Use Committee of Soonchunhyang University Medical School. Specific pathogen-free, 6-week-old female BALB/c mice were used for animal experiments. DEPs (National Institute of Standards and Technology, Gaithersburg, MD, USA) were sterilized by autoclaving and were suspended in saline solution for 30 min before exposure to mice. The DEP-treatment group (n = 8) inhaled 100 μg/m^3^ DEPs via an ultrasonic nebulizer (NE-UO7; Omron Corporation, Tokyo, Japan) for one hour per day, five days a week, for four weeks, with an output of 1 mL/min and 1- to 5-µm particle size^42^. The control group (n = 8) was treated with saline solution under the same conditions as the experimental group. After DEP exposure, animals were sacrificed to gain access to the nasal cavity including the nasal septum and turbinate. The head was separated under anesthesia. After the jaw and nose were removed, nasal tissues including the nasal septum and turbinate between the incisive papilla and second molar were collected.

### Olfactory sensitivity test

The procedure of olfactory sensitivity test was described in our previous study^43^. Before the test, mice were habituated to the testing cage for 30 min. Mice were given distilled water, 10% peanut butter solution, and 10% trichloroacetic acid solution as the control, attractive, and aversive odor, respectively in separate cages (n = 8/group). Each solution was infiltrated into 2 × 2-inch square filter paper, which then was placed on one side of the cage. Mice were introduced to each odor for 3 min, and all sessions were video-recorded. After the test period, mice went through a rest period in another clean, empty cage for 6 min to neutralize the previous odor. The total number and time taken of sniffs were measured after the experiment.

### Microarray

Total RNA from the nasal tissue was extracted using RNeasy Mini kit (Qiagen, Germany) according to the manufacturer’s protocols. Quality control of total RNA was performed using an Agilent 2100 Bioanalyzer (Agilent Technologies, USA). The value of RNA integrity number (RIN) was higher than 8. Microarray was conducted using a Low Input Quick Amp Labeling Kit (Agilent Technologies, USA) following the manufacturer's instructions. Purified cRNA was hybridized on the Mouse Gene Expression Microarray chip 4x44K V2 (ID G2519F-026655; Agilent technologies) with a Gene Expression Hybridization kit (Agilent Technologies, USA), per the manufacturer's instructions. After hybridization, array chips were scanned by the Agilent DNA Microarray Scanner, and signals were quantified using the Feature Extraction Software (Agilent Technologies, USA). Data normalization was analyzed with the GeneSpring Software (Agilent Technologies, USA).

### Network analysis

We identified the list of DEGs from the microarray data on the basis of *p*-value ≤ 0.01 and |fold change| ≥ 2 by using the ExDEGA software version 2.0 (Ebiogen, Korea, https://www.e-biogen.com/). To analyze the signaling network involved in DEP exposure-induced alterations of olfaction, a text mining-based analytical tool, Pathway Studio version 12.1.0.9 (Elsevier, USA, https://www.pathwaystudio.com/) was used. The interactions between DEGs and olfactory-related factors pertaining to the cell, tissue, and organ were visualized and analyzed. We also constructed an expanded pathway containing additional entities such as cellular processes and diseases. To uncover the key genes that contribute significantly to the network, connectivity ratio was considered, which means the percentage of the number of connections a gene has on its network to the number of all connections a gene has on the Pathway Studio database was considered.

### Quantitative real time polymerase chain reaction (qRT-PCR)

The cDNA was synthesized from the extracted RNA using ImProm-II™ Reverse Transcription System (Promega, USA), per the manufacturer’s protocols, and was mixed with SYBR Premix Ex Taq (Takara Bio, Japan). Then, qPCR was performed using Rotor-Gene Q (Qiagen, Germany) per the following thermal cycling conditions: initial denaturation at 95 °C for 5 min, followed by 40 cycles of denaturation at 95 °C for 5 s, annealing at 58 °C for 20 s, and extension at 72 °C for 20 s. The primer sequences are shown in Table S1.

### Statistical analysis

All graph data are expressed as means ± standard deviations (SDs). All experiments were carried out in triplicate. The differences between the control and DEP-treated group were analyzed using the Student's *t* test. *P* values less than 0.05 or 0.01 were considered to indicate statistical significance.

## Supplementary information

Supplementary Tables.

## Data Availability

The microarray data analyzed during the current study are available in the NCBI Gene Expression Omnibus (accession number: GSE114719).
