# Supplementary material for: Genomic approach to explore altered signaling networks of olfaction in response to diesel exhaust particles in mice
Source: Sci Rep. 2020 Oct 12;10:16972. doi: 10.1038/s41598-020-74109-6 (PMC7550584; doi:10.1038/s41598-020-74109-6)
Supplement: Supplementary file 1 — Supplementary Tables. [file 41598_2020_74109_MOESM1_ESM.docx]

**Genomic approach to explore altered signaling networks of olfaction in response to diesel exhaust particles in mice**

Su Ji Kim ^1^, Nahyun Kim ^2^, So Hyeon Park ^2^, Hyun Soo Kim ^1^, Jae-Jun Song ^3^, Bu-Soon Son ^4^, An-Soo Jang ^5^, Moo Kyun Park ^2*^, and Young Rok Seo ^1*^

^1^ Institute of Environmental Medicine for Green Chemistry, Department of Life Science, Dongguk University Biomedi Campus, Goyang-si, Gyeonggi-do, Republic of Korea

^2^ Department of Otorhinolaryngology-Head and Neck Surgery, Seoul National University Hospital, Seoul National University College of Medicine, Seoul, South Korea

^3^ Department of Otorhinolaryngology-Head and Neck Surgery, Korea University College of Medicine, Seoul, South Korea

^4^ Department of Environmental Health Science, Soonchunhyang University, Asan, Republic of Korea

^5^ Division of Allergy and Respiratory Medicine, Department of Internal Medicine, Soonchunhyang University Bucheon Hospital, Bucheon, South Korea

* Corresponding author

Moo Kyun Park, Department of Otorhinolaryngology, Head & Neck Surgery, Seoul National University Hospital, Seoul National University College of Medicine, 101 Daehak-Ro Jongno-Gu, Seoul, 03080, Republic of Korea. E-mail: [aseptic@snu.ac.kr](mailto:aseptic@snu.ac.kr)

Young Rok Seo, Institute of Environmental Medicine for Green Chemistry, Department of Life Science, Dongguk University Biomedi Campus, 32 Dongguk-ro, Ilsandong-gu, Goyang-si, Gyeonggi-do 10326, Republic of Korea. E-mail: [seoyr@dongguk.edu](mailto:seoyr@dongguk.edu)

**Supplementary Information**

**Table S1. Sequences of primers used in qRT-PCR**

| **Gene** | **Forward (5’→3’)** | **Reverse (5’→3’)** |
| --- | --- | --- |
| *Cfap69* | AAACTTACCGGGCTTATCTG | CTGCTCTCTCTGCTTATGTG |
| *Cyp26b1* | AAAAGAGACCTCGCCTCA | AGACTACATCACCCATTAATGCTA |
| *Gapdh* | GAGAAACCTGCCAAGTATG | GTTGCTGTAGCCGTATTC |
| *Il1b* | AGTTGACGGACCCCAAAAGAT | GTTGATGTGCTGCTGCGAGA |
| *Il6* | CTTCCATCCAGTTGCCTTCTTG | AATTAAGCCTCCGACTTGTGAAG |
| *Synpr* | TGACTGATAACGTCCAATGA | AGAACCCTCTTTGGGAATAG |

**Table S2. Differentially expressed genes by DEPs exposure in mouse nasal cavity.**

| **Name** | **Description** |
| --- | --- |
| *Up-regulated genes* |  |
| ACRBP | acrosin binding protein |
| ACSM1 | acyl-CoA synthetase medium chain family member 1 |
| ADGRF1 | adhesion G protein-coupled receptor F1 |
| ADRA1B | adrenoceptor alpha 1B |
| ADRA2B | adrenoceptor alpha 2B |
| AIRN | antisense of IGF2R non-protein coding RNA |
| ALMS1 | ALMS1, centrosome and basal body associated protein |
| AMN1 | antagonist of mitotic exit network 1 homolog |
| ANKK1 | ankyrin repeat and kinase domain containing 1 |
| ANO6 | anoctamin 6 |
| AP5Z1 | adaptor related protein complex 5 subunit zeta 1 |
| ARHGAP17 | Rho GTPase activating protein 17 |
| ARHGAP26 | Rho GTPase activating protein 26 |
| ARHGEF15 | Rho guanine nucleotide exchange factor 15 |
| ARID2 | AT-rich interaction domain 2 |
| ARID3B | AT-rich interaction domain 3B |
| ARL6IP6 | ADP ribosylation factor like GTPase 6 interacting protein 6 |
| ASCL2 | achaete-scute family bHLH transcription factor 2 |
| ASGR1 | asialoglycoprotein receptor 1 |
| ATP8A1 | ATPase phospholipid transporting 8A1 |
| ATXN7 | ataxin 7 |
| BCL2L15 | BCL2 like 15 |
| BIN3 | bridging integrator 3 |
| BPIFC | BPI fold containing family C |
| BSCL2 | BSCL2, seipin lipid droplet biogenesis associated |
| BTN1A1 | butyrophilin subfamily 1 member A1 |
| BZW2 | basic leucine zipper and W2 domains 2 |
| C2CD3 | C2 calcium dependent domain containing 3 |
| CAMK2A | calcium/calmodulin dependent protein kinase II alpha |
| CARNS1 | carnosine synthase 1 |
| CARS2 | cysteinyl-tRNA synthetase 2, mitochondrial |
| CASP14 | caspase 14 |
| CCDC13 | coiled-coil domain containing 13 |
| CCDC138 | coiled-coil domain containing 138 |
| CCDC144B | coiled-coil domain containing 144B (pseudogene) |
| CCDC187 | coiled-coil domain containing 187 |
| CD22 | CD22 molecule |
| CD80 | CD80 molecule |
| CDH4 | cadherin 4 |
| CEP170B | centrosomal protein 170B |
| CEP250 | centrosomal protein 250 |
| CFAP100 | cilia and flagella associated protein 100 |
| CFAP43 | cilia and flagella associated protein 43 |
| CFAP46 | cilia and flagella associated protein 46 |
| CFAP69 | cilia and flagella associated protein 69 |
| CLCN2 | chloride voltage-gated channel 2 |
| CNGB1 | cyclic nucleotide gated channel beta 1 |
| CNNM3 | cyclin and CBS domain divalent metal cation transport mediator 3 |
| CORO2A | coronin 2A |
| CREB5 | cAMP responsive element binding protein 5 |
| CSF3R | colony stimulating factor 3 receptor |
| CYP26B1 | cytochrome P450 family 26 subfamily B member 1 |
| CYP2U1 | cytochrome P450 family 2 subfamily U member 1 |
| DCAF12L2 | DDB1 and CUL4 associated factor 12 like 2 |
| DENND4A | DENN domain containing 4A |
| DENND5B | DENN domain containing 5B |
| DENND6B | DENN domain containing 6B |
| DMBX1 | diencephalon/mesencephalon homeobox 1 |
| DNAJC19 | DnaJ heat shock protein family (Hsp40) member C19 |
| DPYSL5 | dihydropyrimidinase like 5 |
| DSCAML1 | DS cell adhesion molecule like 1 |
| E2F7 | E2F transcription factor 7 |
| EBF4 | early B cell factor 4 |
| EFCAB6 | EF-hand calcium binding domain 6 |
| EPHA5 | EPH receptor A5 |
| EPHB3 | EPH receptor B3 |
| ERMN | ermin |
| ESPN | espin |
| ESR2 | estrogen receptor 2 |
| EXOC6B | exocyst complex component 6B |
| EYA1 | EYA transcriptional coactivator and phosphatase 1 |
| F9 | coagulation factor IX |
| FAM160B2 | family with sequence similarity 160 member B2 |
| FAM219A | family with sequence similarity 219 member A |
| FAM234B | family with sequence similarity 234 member B |
| FAM53B | family with sequence similarity 53 member B |
| FIRRE | firre intergenic repeating RNA element |
| FMO3 | flavin containing monooxygenase 3 |
| FNBP1 | formin binding protein 1 |
| FRMD4B | FERM domain containing 4B |
| FRMPD2 | FERM and PDZ domain containing 2 |
| FRYL | FRY like transcription coactivator |
| GADL1 | glutamate decarboxylase like 1 |
| GALNT6 | polypeptide N-acetylgalactosaminyltransferase 6 |
| GAS5 | growth arrest specific 5 |
| GBP5 | guanylate binding protein 5 |
| GFRA4 | GDNF family receptor alpha 4 |
| GK5 | glycerol kinase 5 |
| GLRX2 | glutaredoxin 2 |
| GNAZ | G protein subunit alpha z |
| GOLGA7B | golgin A7 family member B |
| GTF2I | general transcription factor IIi |
| GXYLT1 | glucoside xylosyltransferase 1 |
| GXYLT2 | glucoside xylosyltransferase 2 |
| H1FOO | H1 histone family member O oocyte specific |
| HECW1 | HECT, C2 and WW domain containing E3 ubiquitin protein ligase 1 |
| HEMGN | hemogen |
| HJURP | Holliday junction recognition protein |
| HLCS | holocarboxylase synthetase |
| HOXB6 | homeobox B6 |
| HRH4 | histamine receptor H4 |
| IFIT1 | interferon induced protein with tetratricopeptide repeats 1 |
| IL15RA | interleukin 15 receptor subunit alpha |
| IP6K2 | inositol hexakisphosphate kinase 2 |
| ITGAM | integrin subunit alpha M |
| ITSN1 | intersectin 1 |
| IVNS1ABP | influenza virus NS1A binding protein |
| KALRN | kalirin RhoGEF kinase |
| KCNA4 | potassium voltage-gated channel subfamily A member 4 |
| KCNJ1 | potassium voltage-gated channel subfamily J member 1 |
| KCNJ5 | potassium voltage-gated channel subfamily J member 5 |
| KCTD14 | potassium channel tetramerization domain containing 14 |
| KDM5B | lysine demethylase 5B |
| KIAA0556 | KIAA0556 |
| KIAA1549L | KIAA1549 like |
| KIF19 | kinesin family member 19 |
| KIF28P | kinesin family member 28, pseudogene |
| KRT15 | keratin 15 |
| KRT19 | keratin 19 |
| LDLRAD3 | low density lipoprotein receptor class A domain containing 3 |
| LGALS4 | galectin 4 |
| LPO | lactoperoxidase |
| LRGUK | leucine rich repeats and guanylate kinase domain containing |
| LRRC43 | leucine rich repeat containing 43 |
| LYPD6 | LY6/PLAUR domain containing 6 |
| LYST | lysosomal trafficking regulator |
| m_1010001N08Rik | RIKEN cDNA 1010001N08 gene |
| m_1500032P08Rik | RIKEN cDNA 1500032P08 gene |
| m_1700022A21Rik | glycerol-3-phosphate dehydrogenase 1-like pseudogene |
| m_1700110I01Rik | RIKEN cDNA 1700110I01 gene |
| m_1810020O05Rik | Riken cDNA 1810020O05 gene |
| m_2010007H06Rik | predicted gene, 32819 |
| m_2010016I18Rik | RIKEN cDNA 2010016I18 gene |
| m_2310002F09Rik | RIKEN cDNA 2310002F09 gene |
| m_2410087M07Rik | RIKEN cDNA 2410087M07 gene |
| m_2610319H10Rik | RIKEN cDNA 2610319H10 gene |
| m_2900055J20Rik | RIKEN cDNA 2900055J20 gene |
| m_3300002I08Rik | zinc finger protein 120-like |
| m_4633401B06Rik | RIKEN cDNA 4633401B06 gene |
| m_4731419I09Rik | RIKEN cDNA 4731419I09 gene |
| m_4731420N21 | uncharacterized protein 4731420N21 |
| m_4732423E21Rik | RIKEN cDNA 4732423E21 gene |
| m_4732440D04Rik | RIKEN cDNA 4732440D04 gene |
| m_4833412C15Rik | RIKEN cDNA 4833412C15 gene |
| m_4930512M02Rik | RIKEN cDNA 4930512M02 gene |
| m_4930590G02Rik | RIKEN cDNA 4921529L05 gene |
| m_4931406E20Rik | RIKEN cDNA 4931406E20 gene |
| m_4933427D06Rik | proline rich 20E |
| m_5230400M03Rik | RIKEN cDNA 5230400M03 gene |
| m_5330406M23Rik | RIKEN cDNA 5330406M23 gene |
| m_5330431K02Rik | RIKEN cDNA 5330431K02 gene |
| m_5330439K02Rik | RIKEN cDNA 5330439K02 gene |
| m_5430434G16Rik | RIKEN cDNA 5430434G16 gene |
| m_5830427D02Rik | RIKEN cDNA 5830427D02 gene |
| m_6330415G19Rik | RIKEN cDNA 6330415G19 gene |
| m_6720462K09Rik | RIKEN cDNA 6720462K09 gene |
| m_6720475J19Rik | RIKEN cDNA 6720475J19 gene |
| m_8030425K09Rik | RIKEN cDNA 8030425K09 gene |
| m_9030419F21Rik | RIKEN cDNA 9030419F21 gene |
| m_9430013L14Rik | RIKEN cDNA 9430013L14 gene |
| m_9530002B09Rik | RIKEN cDNA 9530002B09 gene |
| m_9530083O12Rik | RIKEN cDNA 9530083O12 gene |
| m_9630010G10Rik | RIKEN cDNA 9630010G10 gene |
| m_A130077B15Rik | RIKEN cDNA A130077B15 gene |
| m_A130088B03Rik | RIKEN cDNA A130088B03 gene |
| m_A330023F24Rik | RIKEN cDNA A330023F24 gene |
| m_A430088P11Rik | RIKEN cDNA A430088P11 gene |
| m_A430102J17Rik | RIKEN cDNA A430102J17 gene |
| m_A530006G24Rik | RIKEN cDNA A530006G24 gene |
| m_A730089K16Rik | RIKEN cDNA A730089K16 gene |
| m_AI225934 | expressed sequence AI225934 |
| m_AI605517 | expressed sequence AI605517 |
| m_Akr1b7 | aldo-keto reductase family 1, member B7 |
| m_B230334C09Rik | RIKEN cDNA B230334C09 gene |
| m_B930025B16Rik | RIKEN cDNA B930025B16 gene |
| m_BB166591 | expressed sequence BB166591 |
| m_C030005K06Rik | RIKEN cDNA C030005K06 gene |
| m_C030037D09Rik | RIKEN cDNA C030037D09 gene |
| m_C130068B02Rik | RIKEN cDNA C130068B02 gene |
| m_C230057M02Rik | RIKEN cDNA C230057M02 gene |
| m_C330021F23Rik | RIKEN cDNA C330021F23 gene |
| m_C530014P21Rik | RIKEN cDNA C530014P21 gene |
| m_C81189 | expressed sequence C81189 |
| m_Clca3b | chloride channel calcium activated 4-like |
| m_D030002E05Rik | RIKEN cDNA D030002E05 gene |
| m_D10Bwg1070e | DNA segment, Chr 10, Brigham & Women's Genetics 1070 expressed |
| m_D130012P04Rik | RIKEN cDNA D130012P04 gene |
| m_D130062J21Rik | RIKEN cDNA D130062J21 gene |
| m_D230019N24Rik | RIKEN cDNA D230019N24 gene |
| m_D230034L24Rik | RIKEN cDNA D230034L24 gene |
| m_D230044B12Rik | RIKEN cDNA D230044B12 gene |
| m_D4Ertd681e | DNA segment, Chr 4, ERATO Doi 681, expressed |
| m_D8Ertd158e | DNA segment, Chr 8, ERATO Doi 158, expressed |
| m_D9Wsu90e | DNA segment, Chr 9, Wayne State University 90, expressed |
| m_Defa-rs10 | defensin, alpha, related sequence 10 |
| m_Erdr1 | erythroid differentiation regulator 1 |
| m_Esp6 | exocrine gland secreted peptide 6 |
| m_Gdap10 | ganglioside-induced differentiation-associated-protein 10 |
| m_Gm10087 | predicted gene 10087 |
| m_Gm10405 | predicted gene 10405 |
| m_Gm10432 | predicted gene 10432 |
| m_Gm10576 | predicted gene 10576 |
| m_Gm10914 | predicted gene 10914 |
| m_Gm11213 | predicted gene 11213 |
| m_Gm11412 | predicted gene 11412 |
| m_Gm12576 | predicted gene 12576 |
| m_Gm12887 | predicted gene 12887 |
| m_Gm12888 | predicted gene 12888 |
| m_Gm14135 | predicted gene 14135 |
| m_Gm14496 | predicted gene 14496 |
| m_Gm1553 | predicted gene 1553 |
| m_Gm17753 | predicted gene, 17753 |
| m_Gm3264 | predicted gene 3264 |
| m_Gm3373 | predicted gene 3373 |
| m_Gm35287 | predicted gene, 35287 |
| m_Gm35410 | predicted gene, 35410 |
| m_Gm3858 | predicted gene 3858 |
| m_Gm39553 | predicted gene, 39553 |
| m_Gm41788 | predicted gene, 41788 |
| m_Gm42375 | predicted gene, 42375 |
| m_Gm4470 | predicted gene 4470 |
| m_Gm5878 | predicted gene 5878 |
| m_Gm5938 | predicted gene 5938 |
| m_Gm6252 | predicted gene 6252 |
| m_Gm6981 | glyceraldehyde-3-phosphate dehydrogenase pseudogene |
| m_Gm6994 | predicted gene 6994 |
| m_Gm8479 | thiopurine S-methyltransferase pseudogene |
| m_Gm8893 | serine (or cysteine) preptidase inhibitor, clade A, member 1B pseudogene |
| m_Gm9258 | predicted gene 9258 |
| m_Gm9573 | predicted gene 9573 |
| m_Gm9802 | predicted gene 9802 |
| m_I730030J21Rik | RIKEN cDNA I730030J21 gene |
| m_Lncpint | long non-protein coding RNA, Trp53 induced transcript |
| m_LOC102632383 | zinc finger protein 93-like |
| m_LOC102635992 | major allergen I polypeptide chain 1 |
| m_LOC102641351 | uncharacterized LOC102641351 |
| m_LOC105243194 | uncharacterized LOC105243194 |
| m_LOC105243204 | uncharacterized LOC105243204 |
| m_LOC553096 | uncharacterized LOC553096 |
| m_Mettl7a2 | methyltransferase like 7A2 |
| m_Ms4a4c | membrane-spanning 4-domains, subfamily A, member 4C |
| m_Nctc1 | non-coding transcript 1 |
| m_Olfr1043 | olfactory receptor 481 |
| m_Olfr1348 | olfactory receptor 4 |
| m_Olfr1372-ps1 | olfactory receptor 1372, pseudogene 1 |
| m_Olfr1382 | olfactory receptor 1382 |
| m_Olfr1384 | olfactory receptor 1397 |
| m_Olfr141 | olfactory receptor 516 |
| m_Pitpnm2os1 | phosphatidylinositol transfer protein, membrane-associated 2, opposite strand 1 |
| m_Ppnr | per-pentamer repeat gene |
| m_Rnu1b6 | U1b6 small nuclear RNA |
| m_Scgb1b19 | secretoglobin, family 1B, member 19 |
| m_Scgb1b2 | secretoglobin, family 1B, member 2 |
| m_Scgb1b20 | secretoglobin, family 1B, member 20 |
| m_Scgb1b24 | secretoglobin, family 1B, member 24 |
| m_Scgb1b30 | secretoglobin, family 1B, member 30 |
| m_Scgb1b7 | secretoglobin, family 1B, member 7 |
| m_Scgb2a2 | secretoglobin, family 2A, member 2 |
| m_Scgb2b15 | secretoglobin, family 2B, member 15 |
| m_Scgb2b20 | secretoglobin, family 2B, member 20 |
| m_Scgb2b24 | secretoglobin, family 2B, member 24 |
| m_Scgb2b27 | secretoglobin, family 2B, member 27 |
| m_Scgb2b7 | secretoglobin, family 2B, member 7 |
| m_Six3os1 | SIX homeobox 3, opposite strand 1 |
| m_Skint7 | selection and upkeep of intraepithelial T cells 8 |
| m_Slc2a4rg-ps | Slc2a4 regulator, pseudogene |
| m_Sval2 | seminal vesicle antigen-like 2 |
| m_Tas2r109 | taste receptor, type 2, member 109 |
| m_Vmn2r121 | vomeronasal 2, receptor 121 |
| m_Vmn2r33 | vomeronasal 2, receptor 33 |
| m_Wfdc15b | WAP four-disulfide core domain 15B |
| m_Zfp133-ps | zinc finger protein 133, pseudogene |
| m_Zfp442 | zinc finger protein 442 |
| MAB21L4 | mab-21 like 4 |
| MANSC4 | MANSC domain containing 4 |
| MAP1B | microtubule associated protein 1B |
| MAP6D1 | MAP6 domain containing 1 |
| MAPK15 | mitogen-activated protein kinase 15 |
| MARCH2 | membrane associated ring-CH-type finger 2 |
| MARCH3 | membrane associated ring-CH-type finger 3 |
| MCAM | melanoma cell adhesion molecule |
| MED16 | mediator complex subunit 16 |
| MEDAG | mesenteric estrogen dependent adipogenesis |
| MEF2A | myocyte enhancer factor 2A |
| MLXIPL | MLX interacting protein like |
| MMS22L | MMS22 like, DNA repair protein |
| MRGPRG | MAS related GPR family member G |
| MTFR2 | mitochondrial fission regulator 2 |
| MTIF2 | mitochondrial translational initiation factor 2 |
| MUC4 | mucin 4, cell surface associated |
| MUC5AC | mucin 5AC, oligomeric mucus/gel-forming |
| MUCL1 | mucin like 1 |
| MYT1L | myelin transcription factor 1 like |
| NCBP3 | nuclear cap binding subunit 3 |
| NDOR1 | NADPH dependent diflavin oxidoreductase 1 |
| NEURL1B | neuralized E3 ubiquitin protein ligase 1B |
| NFASC | neurofascin |
| NOC3L | NOC3 like DNA replication regulator |
| NR2E1 | nuclear receptor subfamily 2 group E member 1 |
| NRXN2 | neurexin 2 |
| OASL | 2'-5'-oligoadenylate synthetase like |
| OGA | O-GlcNAcase |
| OGFOD1 | 2-oxoglutarate and iron dependent oxygenase domain containing 1 |
| PAK4 | p21 (RAC1) activated kinase 4 |
| PCLAF | PCNA clamp associated factor |
| PCSK4 | proprotein convertase subtilisin/kexin type 4 |
| PDE4C | phosphodiesterase 4C |
| PDE7B | phosphodiesterase 7B |
| PER1 | period circadian regulator 1 |
| PIRT | phosphoinositide interacting regulator of transient receptor potential channels |
| PLCB4 | phospholipase C beta 4 |
| PLEKHG3 | pleckstrin homology and RhoGEF domain containing G3 |
| PLPBP | pyridoxal phosphate binding protein |
| POLR2C | RNA polymerase II subunit C |
| POT1 | protection of telomeres 1 |
| PPRC1 | peroxisome proliferator-activated receptor gamma, coactivator-related 1 |
| PRKN | parkin RBR E3 ubiquitin protein ligase |
| PRR36 | proline rich 36 |
| PRR7 | proline rich 7, synaptic |
| PRSS37 | serine protease 37 |
| PSME3 | proteasome activator subunit 3 |
| PTPDC1 | protein tyrosine phosphatase domain containing 1 |
| RALGPS1 | Ral GEF with PH domain and SH3 binding motif 1 |
| RARB | retinoic acid receptor beta |
| RBM33 | RNA binding motif protein 33 |
| RGS11 | regulator of G protein signaling 11 |
| RNF103 | ring finger protein 103 |
| RNF165 | ring finger protein 165 |
| RNF224 | ring finger protein 224 |
| ROBO2 | roundabout guidance receptor 2 |
| SACS | sacsin molecular chaperone |
| SCAF8 | SR-related CTD associated factor 8 |
| SCN8A | sodium voltage-gated channel alpha subunit 8 |
| SEMA4C | semaphorin 4C |
| SEMA6D | semaphorin 6D |
| SEPT3 | septin 3 |
| SERPINA1 | serpin family A member 1 |
| SERPINB8 | serpin family B member 8 |
| SFI1 | SFI1 centrin binding protein |
| SGSH | N-sulfoglucosamine sulfohydrolase |
| SHANK1 | SH3 and multiple ankyrin repeat domains 1 |
| SHLD2 | shieldin complex subunit 2 |
| SLC10A1 | solute carrier family 10 member 1 |
| SLC1A4 | solute carrier family 1 member 4 |
| SLC25A27 | solute carrier family 25 member 27 |
| SLC26A4 | solute carrier family 26 member 4 |
| SLC26A7 | solute carrier family 26 member 7 |
| SLC35D1 | solute carrier family 35 member D1 |
| SLC4A8 | solute carrier family 4 member 8 |
| SLC4A9 | solute carrier family 4 member 9 |
| SLFN13 | schlafen family member 13 |
| SNHG11 | small nucleolar RNA host gene 11 |
| SPAG7 | sperm associated antigen 7 |
| SPTBN4 | spectrin beta, non-erythrocytic 4 |
| SREK1 | splicing regulatory glutamic acid and lysine rich protein 1 |
| ST6GALNAC4 | ST6 N-acetylgalactosaminide alpha-2,6-sialyltransferase 4 |
| SUV39H2 | suppressor of variegation 3-9 homolog 2 |
| SYNPO2L | synaptopodin 2 like |
| TBC1D30 | TBC1 domain family member 30 |
| TBX6 | T-box 6 |
| TCF7L2 | transcription factor 7 like 2 |
| TENM2 | teneurin transmembrane protein 2 |
| THOC7 | THO complex 7 |
| TIA1 | TIA1 cytotoxic granule associated RNA binding protein |
| TKFC | triokinase and FMN cyclase |
| TMC6 | transmembrane channel like 6 |
| TMEM145 | transmembrane protein 145 |
| TMEM239 | transmembrane protein 239 |
| TMEM94 | transmembrane protein 94 |
| TNFRSF4 | TNF receptor superfamily member 4 |
| TNFRSF9 | TNF receptor superfamily member 9 |
| TRIM25 | tripartite motif containing 25 |
| TSHZ2 | teashirt zinc finger homeobox 2 |
| TSKU | tsukushi, small leucine rich proteoglycan |
| TTC21A | tetratricopeptide repeat domain 21A |
| TTC38 | tetratricopeptide repeat domain 38 |
| TTLL10 | tubulin tyrosine ligase like 10 |
| TTLL3 | tubulin tyrosine ligase like 3 |
| TTLL6 | tubulin tyrosine ligase like 6 |
| TULP1 | tubby like protein 1 |
| UBE2I | ubiquitin conjugating enzyme E2 I |
| UBN2 | ubinuclein 2 |
| UBTF | upstream binding transcription factor |
| UBXN10 | UBX domain protein 10 |
| UNC13C | unc-13 homolog C |
| UNG | uracil DNA glycosylase |
| VAMP5 | vesicle associated membrane protein 5 |
| VAX2 | ventral anterior homeobox 2 |
| VPS35L | VPS35 endosomal protein sorting factor like |
| WDR89 | WD repeat domain 89 |
| YBX2 | Y-box binding protein 2 |
| ZCCHC9 | zinc finger CCHC-type containing 9 |
| ZDHHC6 | zinc finger DHHC-type containing 6 |
| ZNF488 | zinc finger protein 488 |
| ZNF76 | zinc finger protein 76 |
| ZNF791 | zinc finger protein 791 |
| ZNF93 | zinc finger protein 93 |
| *Down-regulated genes* |  |
| ABHD5 | abhydrolase domain containing 5 |
| ACOXL | acyl-CoA oxidase like |
| ACSS3 | acyl-CoA synthetase short chain family member 3 |
| ADA | adenosine deaminase |
| ADAMTS4 | ADAM metallopeptidase with thrombospondin type 1 motif 4 |
| AMBN | ameloblastin |
| AMELX | amelogenin X-linked |
| ARNTL | aryl hydrocarbon receptor nuclear translocator like |
| ASAH2 | N-acylsphingosine amidohydrolase 2 |
| ATL2 | atlastin GTPase 2 |
| AZIN1 | antizyme inhibitor 1 |
| B3GNT5 | UDP-GlcNAc:betaGal beta-1,3-N-acetylglucosaminyltransferase 5 |
| C2CD4B | C2 calcium dependent domain containing 4B |
| C5orf46 | chromosome 5 open reading frame 46 |
| CAMSAP2 | calmodulin regulated spectrin associated protein family member 2 |
| CASP4 | caspase 4 |
| CCL17 | C-C motif chemokine ligand 17 |
| CCL2 | C-C motif chemokine ligand 2 |
| CCNB1 | cyclin B1 |
| CCNB2 | cyclin B2 |
| CCNG2 | cyclin G2 |
| CDC20 | cell division cycle 20 |
| CDK7 | cyclin dependent kinase 7 |
| CEBPD | CCAAT enhancer binding protein delta |
| CLEC2D | C-type lectin domain family 2 member D |
| CNFN | cornifelin |
| COL11A1 | collagen type XI alpha 1 chain |
| COL12A1 | collagen type XII alpha 1 chain |
| COL3A1 | collagen type III alpha 1 chain |
| CRCT1 | cysteine rich C-terminal 1 |
| CREM | cAMP responsive element modulator |
| CRISP3 | cysteine rich secretory protein 3 |
| CRISPLD2 | cysteine rich secretory protein LCCL domain containing 2 |
| CXCL1 | C-X-C motif chemokine ligand 1 |
| CYSRT1 | cysteine rich tail 1 |
| DCT | dopachrome tautomerase |
| DEFB114 | defensin beta 114 |
| DSPP | dentin sialophosphoprotein |
| DUPD1 | dual specificity phosphatase and pro isomerase domain containing 1 |
| ECT2 | epithelial cell transforming 2 |
| ELOVL1 | ELOVL fatty acid elongase 1 |
| ENAM | enamelin |
| ENDOU | endonuclease, poly(U) specific |
| GAD1 | glutamate decarboxylase 1 |
| GBP2 | guanylate binding protein 2 |
| GJA1 | gap junction protein alpha 1 |
| GSTA5 | glutathione S-transferase alpha 5 |
| HACD2 | 3-hydroxyacyl-CoA dehydratase 2 |
| HILPDA | hypoxia inducible lipid droplet associated |
| IDI2 | isopentenyl-diphosphate delta isomerase 2 |
| IL1B | interleukin 1 beta |
| IL20 | interleukin 20 |
| IL6 | interleukin 6 |
| KLF10 | Kruppel like factor 10 |
| KRT13 | keratin 13 |
| KRT16 | keratin 16 |
| KRT24 | keratin 24 |
| KRT32 | keratin 32 |
| KRT4 | keratin 4 |
| KRTDAP | keratinocyte differentiation associated protein |
| LACTB2 | lactamase beta 2 |
| LAMC2 | laminin subunit gamma 2 |
| LCE3B | late cornified envelope 3B |
| LCE3E | late cornified envelope 3E |
| LRRC17 | leucine rich repeat containing 17 |
| LUM | lumican |
| LYG1 | lysozyme g1 |
| m_1200007C13Rik | RIKEN cDNA 1200007C13 gene |
| m_2310057J18Rik | similar to RIKEN cDNA 2310057J18 |
| m_Acot5 | acyl-CoA thioesterase 5 |
| m_BC037156 | cDNA sequence BC037156 |
| m_Gm4788 | predicted gene 4788 |
| m_Lbhd2 | LBH domain containing 2 |
| m_Mup8 | major urinary protein 8 |
| m_Olfr1135 | olfactory receptor 578 |
| m_Retnlg | resistin-like gamma |
| m_Scgb2b15 | secretoglobin, family 2B, member 15 |
| m_Serpina3f | serine (or cysteine) peptidase inhibitor, clade A, member 3F |
| m_Serpinb3c | serine (or cysteine) peptidase inhibitor, clade B, member 3C |
| m_Sprr2a2 | small proline-rich protein 2A2 |
| m_Sprr2h | small proline-rich protein 2H |
| m_Stfa2l1 | stefin A2 like 1 |
| m_Sva | seminal vesicle antigen |
| m_Teddm3 | similar to RIKEN cDNA 2310042E22 |
| m_U90926 | cDNA sequence U90926 |
| m_Wfdc21 | WAP four-disulfide core domain 21 |
| MAB21L3 | mab-21 like 3 |
| MMP13 | matrix metallopeptidase 13 |
| MMP3 | matrix metallopeptidase 3 |
| NABP1 | nucleic acid binding protein 1 |
| NAT8L | N-acetyltransferase 8 like |
| NUDT7 | nudix hydrolase 7 |
| ODAM | odontogenic, ameloblast associated |
| PCK1 | phosphoenolpyruvate carboxykinase 1 |
| PHEX | phosphate regulating endopeptidase homolog X-linked |
| PITX2 | paired like homeodomain 2 |
| PLAT | plasminogen activator, tissue type |
| PLOD2 | procollagen-lysine,2-oxoglutarate 5-dioxygenase 2 |
| POSTN | periostin |
| PPBP | pro-platelet basic protein |
| PRR13 | proline rich 13 |
| RARRES1 | retinoic acid receptor responder 1 |
| RGS8 | regulator of G protein signaling 8 |
| S100A9 | S100 calcium binding protein A9 |
| SCD | stearoyl-CoA desaturase |
| SCIN | scinderin |
| SERPINB10 | serpin family B member 10 |
| SERPINB12 | serpin family B member 12 |
| SERPINB4 | serpin family B member 4 |
| SGMS2 | sphingomyelin synthase 2 |
| SIM2 | SIM bHLH transcription factor 2 |
| SLC22A4 | solute carrier family 22 member 4 |
| SLC26A1 | solute carrier family 26 member 1 |
| SLC7A11 | solute carrier family 7 member 11 |
| SLPI | secretory leukocyte peptidase inhibitor |
| SPARC | secreted protein acidic and cysteine rich |
| SPON2 | spondin 2 |
| SPRR2D | small proline rich protein 2D |
| STEAP4 | STEAP4 metalloreductase |
| SYNPR | synaptoporin |
| TAC1 | tachykinin precursor 1 |
| TGM1 | transglutaminase 1 |
| THBS1 | thrombospondin 1 |
| THBS4 | thrombospondin 4 |
| THEM5 | thioesterase superfamily member 5 |
| TIMP1 | TIMP metallopeptidase inhibitor 1 |
| TMED5 | transmembrane p24 trafficking protein 5 |
| TMPRSS11A | transmembrane serine protease 11A |
| TMPRSS11D | transmembrane serine protease 11D |
| TNFAIP2 | TNF alpha induced protein 2 |
| TNFRSF12A | TNF receptor superfamily member 12A |
| TUBB6 | tubulin beta 6 class V |
| VCAM1 | vascular cell adhesion molecule 1 |
| VSIG8 | V-set and immunoglobulin domain containing 8 |
